# Influences of Prejudice and Stereotyping in the Direct Care Workforce

**DOI:** 10.1093/geroni/igab046.3495

**Published:** 2021-12-17

**Authors:** Jennifer May

**Affiliations:** Duke University, Gaston, North Carolina, United States

## Abstract

Direct Care Workers (DCW; nursing assistants, personal care aides, home health aides) have the most one on one care with sexual and gender minority (SGM) older adults who reside in residential care facilities or use home health services. DCWs make up a vast majority of the healthcare workforce, holding almost five million jobs in 2019, with approximately 70% of the positions held being in residential care facilities. In a qualitative design study, 11 DCWs were interviewed using an open-ended, semi-structured format to describe their perceptions of care provided to SGM older adults in residential care facilities and the home health setting. These results were part of a larger qualitative study which found there were cues of stereotyping and prejudice in DCW narratives toward SGM older adults. The category DCWs’ care and social system referred to characteristics of the DCWs’ work environment and the perspectives, attitudes, and reported care toward SGM older adults and diverse populations. It was determined that there are synergies among SGM older adults’ care and DCW along with DCW workforce issues (short staffed, low wages, lack of health benefits) that may prevent the DCW from being accepting of implicit bias training or culture change within these facilities/agencies. Implications for practice, policy, and future research are discussed.

